# Monitoring SARS-CoV-2 Surrogate TGEV Individual Virions Structure Survival under Harsh Physicochemical Environments

**DOI:** 10.3390/cells11111759

**Published:** 2022-05-27

**Authors:** Miguel Cantero, Diego Carlero, Francisco Javier Chichón, Jaime Martín-Benito, Pedro José De Pablo

**Affiliations:** 1 Departamento de Física de la Materia Condensada, Universidad Autónoma de Madrid, 28049 Madrid, Spain; miguel.cantero@uam.es; 2 Departamento de Estructura de Macromoléculas, Centro Nacional de Biotecnología CSIC, 28049 Madrid, Spain; dcarlero@cnb.csic.es (D.C.); fjchichon@cnb.csic.es (F.J.C.); 3 Instituto de Física de la Materia Condensada IFIMAC, Universidad Autónoma de Madrid, 28049 Madrid, Spain

**Keywords:** physical virology, coronavirus, mechanical properties, disinfection, uncoating

## Abstract

Effective airborne transmission of coronaviruses via liquid microdroplets requires a virion structure that must withstand harsh environmental conditions. Due to the demanding biosafety requirements for the study of human respiratory viruses, it is important to develop surrogate models to facilitate their investigation. Here we explore the mechanical properties and nanostructure of transmissible gastroenteritis virus (TGEV) virions in liquid milieu and their response to different chemical agents commonly used as biocides. Our data provide two-fold results on virus stability: First, while particles with larger size and lower packing fraction kept their morphology intact after successive mechanical aggressions, smaller viruses with higher packing fraction showed conspicuous evidence of structural damage and content release. Second, monitoring the structure of single TGEV particles in the presence of detergent and alcohol in real time revealed the stages of gradual degradation of the virus structure in situ. These data suggest that detergent is three orders of magnitude more efficient than alcohol in destabilizing TGEV virus particles, paving the way for optimizing hygienic protocols for viruses with similar structure, such as SARS-CoV-2.

## 1. New concepts

The study of the structural and physicochemical stability of viruses to adverse environments is becoming increasingly relevant to the present or future pandemics. One of the main fronts against the COVID-19 disease are disinfection methods, including hydroalcoholic solutions and detergents. Here we report the mechano-structural stability of individual coronavirus virions using a structurally indistinguishable surrogate, the porcine transmissible gastroenteritis virus (TGEV). We probe virions’ resistance under a range of physicochemical assaults, including mechanical stress, desiccation and treatment with fixative (paraformaldehyde) and biocidal chemical agents (detergent and ethanol). Our experiments reveal that virion stability depends on differences in the packing fraction between virus particles due to their pleomorphic nature. We find that individual viral particles crumble under a detergent concentration three orders of magnitude lower than ethanol. These experiments determined that detergent reduces virus size by 15–30 nm by solubilizing the lipid virus envelope, whereas ethanol dehydrates and shrinks TGEV virions by ~12 nm. Both biocidal agents induced material spreading around virus particles that correspond to externalization of ribonucleoproteins. This work suggests the convenience of using a SARS-CoV2 surrogate for dynamic nanoscale structure studies that can alleviate the use of BSL3 labs that are highly demanded for biomedical and biotechnological research.

## 2. Introduction

Viruses can be considered to be molecular machines that parasitize the host cellular machinery to perpetuate themselves [1]. The virion itself plays several roles in the viral life cycle including self-assembly, packaging and protection of nucleic acid from the extracellular environment during transmission, recognition of host cells and intracellular release of its genome. In a contradictory *tour de force*, virus particles must be stable enough to protect the genome during transmission but prepared to easily release it at the right time and place [2]. 

A major challenge in the current and future pandemic scenarios is to control virus spread [3]. The proposed vector for respiratory viruses such as influenza or severe acute respiratory syndrome coronavirus (SARS-CoV-2) is the ensemble of micrometric droplets [4] emitted through the airways of infected individuals [5,6]. These aerosols can be directly inhaled or deposited on surfaces, which can become fomites responsible for indirect virus transmission. Viral propagation depends on the physicochemical conditions of the environment (humidity, pH, temperature, etc.) being sufficient to maintain functionality of the virus [7]. In the case of SARS-CoV-2, infectivity is maintained from 2 h [8] to more than 28 days [9]. SARS-CoV-1, which emerged in 2003, exhibited similar survival times [8].

When zoonotic animal viruses spread in humans, they can become a serious health threat because the population is immunologically naïve [10,11], leading to global pandemics such as COVID-19 currently. SARS-CoV-2 has shown particularly high transmissibility compared to previous respiratory CoVs, [12] thus any research should be conducted in a BSL-3 environment [13]. To circumvent this limitation, it is possible to inactivate SARS-CoV-2 using chemical fixatives such as paraformaldehyde (PFA) or glutaraldehyde [14,15] while keeping its structure intact. However, these treatments preclude in situ dynamics experiments [16] and also studies of virion stability [17]. SARS-CoV-2 virus-like-particles (VLPs) have also been used as a safe conduit for investigating the stability of the CoV shell [18] but for mechanical studies the capsid-core interaction that is lacking in VLPs is imperative. In this context, it is evident that although biomedical investigation of COVID-19 would ideally be performed with SARS-CoV-2 particles, the search for a surrogate model that allows structural studies to be performed safely outside of BSL3 facilities would be greatly beneficial. We chose the transmissible gastroenteritis virus (TGEV), a human-safe swine CoV [19,20], as one such surrogate model. TGEV and SARS-CoV-2 are both enveloped, single-stranded positive-sense RNA viruses which share most of their structural features. Virions are roughly spherical particles of around 100–160 nm in diameter that contain four structural proteins: spike (S), envelope (E), membrane (M) and nucleoprotein (N). The first three (S, E and M) are membrane related proteins, whereas N protein encapsidates the viral genome forming the ribonucleoprotein (RNP), which fills most of the internal volume of the virion and interacts with M protein on the inner surface of the membrane [21]. All of these common features make the viruses virtually indistinguishable from a structural point of view, making TGEV an excellent surrogate model for the investigation of the dynamics and stability of CoV in general. In fact, TGEV has already been used as a model of SARS-CoV-1 and SARS-CoV-2, especially in studies of astringent environmental conditions for virions and in studies of sensitivity to biocidal agents [22,23,24]. However, these studies did not explore the structural damage of the virions. 

During the fight against COVID-19, hygienic and prophylactic practices have been globally recommended to stop virus spread, such as face masks [25] and disinfection of potential fomites with biocidal agents [9]. Most studies on the presence and survival of viruses on surfaces are verified by detection of viral genomes (PCR) and by titration assays that analyze the infective capacity of the particles (cytopathic effect, expression of viral proteins or formation of progeny) [26]. In virology, studies of the mechano-structural alterations of virions are rarely used to establish a direct interplay between virus infectivity and the structural state, and little is known about the structural determinants of the infectivity at the single molecule level.

In recent years, atomic force microscopy (AFM) has been used to thoroughly characterize the physical properties, structure and stability of many viruses [27]. The core of an AFM consists of a nanoscopic stylus located at the end of a microcantilever that is used as a force transducer to palpate the sample adsorbed on a solid substrate. In this manner, it is possible to scan individual viruses, obtaining their topography and a variety of physical properties such as mechanics [28] or electrostatics [29] in controlled liquid milieu. AFM has provided biophysical information on all kinds of viruses, including bacteriophages [14,30,31,32,33] and eukaryotic viruses [33,34,35,36,37,38,39,40]. With this technique, SARS-CoV-2 has been reported as a relatively soft particle with a highly resilient capacity under mechanical deformation and little thermal stability [16,17,18]. However, little is known about the effect of adverse physicochemical environments on the structure and stability of virus particles at the nanoscale [41], including daily healthcare biocidal agents, such as hydroalcoholic gels and detergents. In fact, previous works did not monitor the viral structure evolution in real time. Although these studies provide useful information about the virion structural integrity, they are limited to static studies in which the initial and final states of the virions are observed, losing the information of the intermediate stages.

Here, we use AFM to explore in real time the stability of individual TGEV particles as a surrogate model for SARS-CoV-2 in order to elucidate its structural stability under a range of physicochemical assaults, including mechanical stress, desiccation-rehydration cycles and treatment with chemical agents commonly used as biocides, such as detergents and ethanol. Likewise, we aim to show that some structural research can be performed with non-hazardous CoV strains. 

## 3. Materials and Methods

### 3.1. Cell Culture and Virus Purification

#### 3.1.1. Cells and Viruses

Swine testis (ST) cells (ATCC, CRL-1746) [42] were grown in Dulbecco’s modified Eagle medium (DMEM) supplemented with 10% fetal bovine serum (FBS). The TGEV PUR46-MAD strain [43] was used to infect ST cells and virus titration was performed on ST cell monolayers as previously described [44].

#### 3.1.2. Purification of TGEV Virions

Initially, supernatants from TGEV-infected cells were clarified by centrifugation at 4500× *g* for 20 min at 4 °C. Virions were then sedimented by ultracentrifugation at 112,000× *g* for 120 min through a 31% (*w*/*w*) sucrose cushion in TEN buffer (10 mM Tris-HCl [pH 7.4], 1 mM EDTA, 1 M NaCl) with 0.2% Tween 20 (Sigma, Saint Louis, MI, USA). The viruses were subsequently centrifuged through a continuous 15% to 42% (*w*/*w*) sucrose gradient in order to separate wild-type virions and defective virions with lower density. Finally, virions were pelleted by ultracentrifugation at 112,000× *g* for 60 min and resuspended in TNE buffer (10 mM Tris-HCl [pH 7.4], 1 mM EDTA, 100 mM NaCl). Purified virions were disaggregated by sonication (six pulses at medium intensity in a Branson sonifier 450). SDS-PAGE protein analysis of the purified TGEV virions using Coomassie staining showed the presence of the major viral proteins S, N and M. This result confirmed the presence of TGEV virions in the samples used for AFM and cryogenic electron microscopy.

### 3.2. Electron Microscopy

Purified TGEV virions were applied to Cu/Rh 300 mesh Quantifoil grids and vitrified by plunge freezing in liquid ethane using a Vitrobot Mark IV (Thermofisher Scientific, Waltham, MA, USA) cryo-fixation unit. The vitrified grids were transferred to a Talos Arctica electron microscope (Thermofisher Scientific, Waltham, MA, USA) operated at 200 kV under cryogenic conditions. The tomographic tilt-series from −60° to 60° at 3° step were recorded on a Falcon III direct detector using TOMOGRAPHY 4 software (Thermofisher Scientific, Waltham, MA, USA) at a nominal magnification of x57K, applying a defocus of ~3 µm and with a sampling ratio of 1.8 Å/pixel. Tilt series were aligned using IMOD software [45] and CTF was corrected by using NovaCTF software [46].

### 3.3. AFM

Muscovite mica treated with poly-L-lysine was used as support substrate. A 30 µL droplet of 0.1% poly-L-lysine was deposited onto freshly cleaved mica for 15 min and rinsed with ultra-pure water 3 times. After every rinse, a highly pure stream of N_2_ was applied to the surface to dry it. Then, a 30 µL droplet with a viral titer of ~2 × 10^6^ virus/mL in TNE buffer was incubated for 40 min and rinsed with a clean buffer 6 times, adding 1× incubation volume and removing it. A final volume of 120 µL was then used for measuring the virus.

All the experiments were carried out with NANOSENSORS™ qp-BioAC AFM probes, with a tip radius smaller than 10 nm. The cantilevers were calibrated with Sader’s method [47] for rectangular cantilevers. The spring constants used for the experiments were 0.05 and 0.1 N/m corresponding to the 80 × 30 µm^2^ and 60 × 25 µm^2^ cantilevers, respectively.

Measurements were performed with an AFM (Nanotec Electrónica S.L., Madrid, Spain) in Jumping Plus Mode [48]. In this mode, lateral interactions are minimized as the lateral displacement is done with the tip far from the sample. The tip moves in the Z axis (perpendicular to the surface) until a certain present interaction force. Topography data is obtained, and the tip releases the surface and is moved laterally one pixel to repeat the procedure again.

For the nanoindentation assays, individual particles were deformed with the AFM tip by performing single force-distance curves (FDC) at a constant speed (50 nm/s). The first and second FDC were performed with a Z piezo displacement of 150 nm. Thereafter, Z piezo displacement was increased to 200 and 300 nm, consecutively. The elastic modulus of the particles was obtained from the linear regime of the FDC curves. Both breaking force and critical indentation were defined as the point at which a major drop in the normal force was obtained, usually after the end of the linear regime, meaning a failure in the integrity of the shell.

All the images and curves were processed and analyzed using the WSxM software [49].

### 3.4. Detergent and Ethanol Time Courses

For the time-course experiments, IGEPAL^®^ CA-630 (Sigma-Aldrich, Saint Louis, MI, USA, CAS: 9002-93-1) was diluted into a TNE buffer to get 1, 0.2 and 0.08% *v*/*v* solutions. This detergent was selected for its non-denaturing properties and well-established protocol for inactivating human CoVs [50]. Ethanol (Ethanol absolute pure, PanReac AppliChem, CAS: 64-17-5) was mixed with TNE 10× buffer to get a TNE 1× 60% ethanol *v*/*v* buffer. Gradients were performed with Genie Syringe Pump (Kent Scientific, Torrington, CT, USA).

## 4. Results

### 4.1. TGEV Structure Exhibits Different Topographies under Diverse Environments

**Figure 1:**
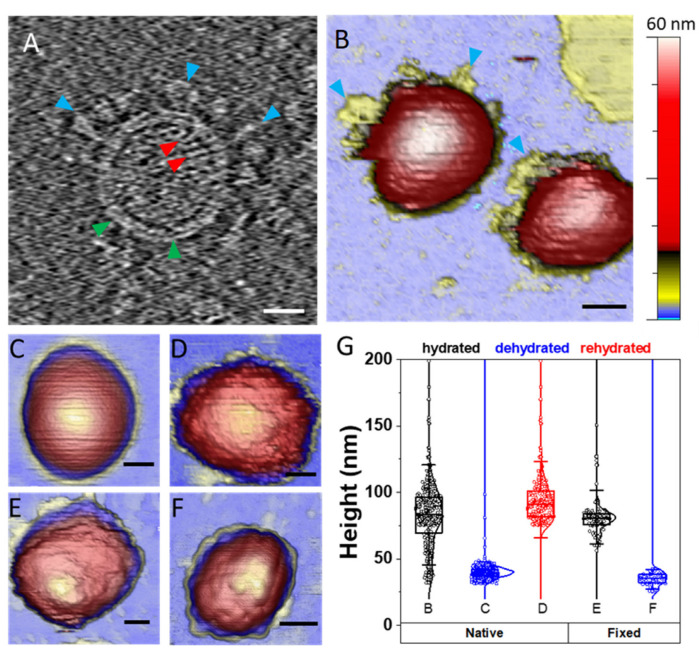
Structural characterization of TGEV virions (**A**) Cryo-electron tomogram presenting a section of a TGEV particle showing the spike proteins (blue arrowheads), the double layer membrane (green arrowheads) and the RNP wrapped inside (red arrowheads). Video S1 shows sections of a reconstructed cryotomogram of a group of purified TGEV virions (**B**) Intact virions visualized by AFM in a buffered solution. Blue arrowheads point to possible spike debris. The color bar shows the arbitrary color palette used for AFM image visualization, from 0 nm (blue) to 60 nm (white). Maximum color is set to the maximum height in each image (**C**) Dehydrated virion imaged in air conditions (**D**) Rehydrated virion imaged in liquid conditions (**E**) PFA-fixed virion imaged in liquid (**F**) Dehydrated PFA-fixed virion imaged in air (**G**) Height chart comparing the five populations with the corresponding AFM images labels. The native and fixed-hydrated populations had the same mean height, although the fixed particles had a narrower height distribution. Dehydration resulted in an average decrease in height of about 50 nm, although rehydration restored the height of native particles. Scale bars represent 50 nm unless otherwise indicated.

After TGEV purification (see the Materials and Methods), the integrity of the virions was checked by cryo-electron tomography, revealing the described morphology of the virus with the ribonucleoproteins (RNPs), membrane and spikes (Figure 1A and Supplementary Video S1). 

For AFM observation, virions were adsorbed on poly-L-lysine functionalized mica to obtain a random distribution of isolated viral particles on the surface under buffered conditions (Figure S1A) and subsequently imaged in detail in different environments. Hydrated native viral particles showed a smooth and curved surface (Figure 1B), as previously described [16,17,51]. To determine the effect of dehydration on the virions structure, the samples were first rinsed with distilled water and then dried once using a stream of N_2_ to remove the liquid from the surface (Figure 1C). Thereafter, the sample was rehydrated and new images were taken (Figure 1D). Likewise, the effect of chemical fixation on virion topology was also studied under similar conditions. For this purpose, samples of PFA-fixed particles were visualized in hydrated (Figure 1E) and dehydrated (Figure 1F) states following the same protocol described in the previous case.

Although characterization of structures with a size similar to the radius of the AFM tip apex, such as a virion, is strongly influenced by dilation artifacts in the lateral dimension [52], vertical measurements are not affected by this limitation and are accurate at the nanometer scale. Therefore, virus height is often used as the first indicator of virus integrity after adsorption on a surface [53]. The hydrated native TGEV particles’ height ranged from ~30 to ~140 nm (Figure 1G), which highlights the pleomorphism of TGEV as described for other CoVs [51,52,53,54], and resulted into two main populations, one at ~43 nm and the highest peak, at ~75 nm (Figure S1B). When virions were dehydrated, they lost around 40% of their height, as shown in previous studies [55], and also presented a smooth surface. In the case of rehydrated particles, they seemed to recover their initial height (Figure 1D), although their surface presented a roughness far different from the initial smooth condition (Figure 1B). Similarly, PFA-fixed virions also displayed a rugged surface no matter the hydration state and presented a narrower height distribution compared with untreated virus particles (Figure 1G), as previously reported with SARS-CoV-2 [16].

### 4.2. TGEV Virions Exhibit Two Stability Behaviors under Mechanical Deformation

**Figure 2:**
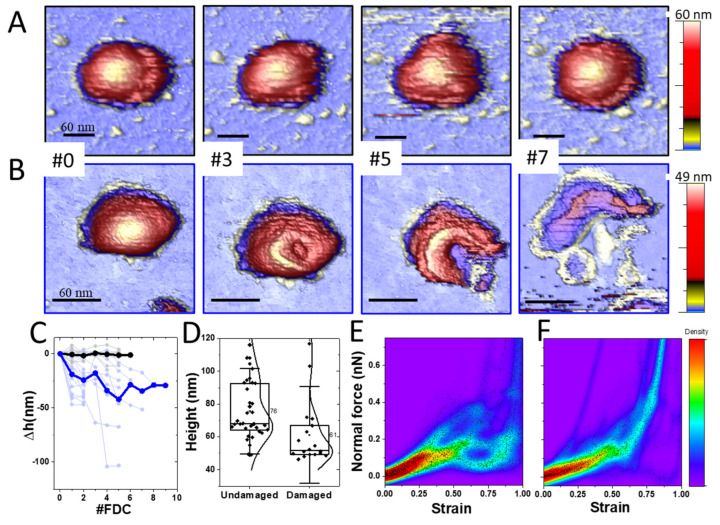
Mechanical characterization of TGEV particles. (**A**,**B**) Topographical images of TGEV virions after consecutive indentations. Particles can be classified in two groups: indentation resistive ((**A**), Video S2) and indentation sensitive ((**B**), Video S3) as shown in the topo images. Frame number indicates how many indentations were performed so far (**C**). Chart evolution of Δheight as a function of the indentation number (FDC#). The values of height are taken from the indented region (**D**). Height plot distribution of particles before the indentation experiments, which agrees with the scale bars of (**A**,**B**,**E**,**F**). The heat map displays all the density of pixels for all the force-distance curves (FDC) of all particles as a function of the strain (Figure S2) for the indentation-resistive (**E**) and the indentation-sensitive (**F**) particles. Reddish colors mean more density of points which points to the coincidence of many experiments.

The resistance of virus particles to mechanical deformation is another indicator of structural stability. For this purpose, we located individual virus particles and performed a nanoindentation with a force vs. distance curve (FDC), which consists of pushing one virus at the very top with the AFM tip while registering the cantilever deflection [2,56]. These experiments (Figure S2), beyond providing information about stiffness and fragility, allow virus manipulation and subsequent monitoring of its structure [36]. Virions were first deformed by displacing the piezoelectric tube holding the sample 150 nm in the Z axis at 50 nm/s. In subsequent deformations, the Z piezo displacement was gradually increased to reach between 200 and 300 nm at the same speed. These experiments unveiled two different behaviors of TGEV: In the first case (Figure 2A), a virus particle was probed seven times with maximum forces ranging from 0.6 nN to 3 nN (Figure S2A), remaining unaltered through the alternating AFM images (Video S2). In fact, ~65% of the explored virus particles (*N* = 69) kept their height constant (Figure 2C, black), indicating that their structure was elastic and not affected by the nanoindentations. However, in the second case (Figure 2B) and using the same FDC number and parameters, the TGEV particle showed evident structural changes (Video S3) consisting of the appearance of a circular crater after the first FDC that enlarged after consecutive deformations until the virus appears to have ripped open, releasing its content (Figure 2B#7). This behavior was observed in ~35% of the explored particles and was accompanied by a gradual decrease in height after each indentation (Figure 2C, blue). It is important to remark that while the mechanical properties of both kinds of particles were similar (Figure S3), the initial height of the particles that remained undamaged (indentation resistive) was larger than the damaged ones (indentation sensitive) (Figure 2D), a difference that was statistically significant at a 95% level of confidence. We also classified the first indentation curve for each virus type (Figure S2), though analysis of these data was hampered by the pleomorphic nature of TGEV since the indentation at a given force for each virus particle depends on its size. Therefore, we normalized each indentation to the strain, from 0 (no deformation) to 1 (maximum indentation) and plotted these data in thermal maps for all particles (Figure 2E,F). In the case of indentation-resistive viruses, the force-strain curve (Figure 2E) revealed a certain homogeneity in the linear regime up to a strain of ~0.5, exhibiting a disordered nature afterwards. However, in the case of indentation-sensitive particles, the data portrayed a homogeneous behavior (Figure 2F). In addition, a comparison of the spring constant, breaking force and critical indentation of both groups showed no significant difference at a 95% level of confidence (Figure S3).

### 4.3. TGEV Structure Is Differently Affected by Detergents and Ethanol

After checking TGEV’s mechanical stability, we investigated the chemical resistance of virions to two biocidal agents that are commonly used for hygiene and disinfection: detergents and ethanol. Ethanol has been proven to be virucidal [57] and is one of the main components in many hydrogels currently used in daily sanitizers to prevent SARS-CoV-2 on surfaces [58]. Similarly, detergents are recommended by the WHO as an effective tool against CoV. Here, we addressed not only the structural changes of the virus associated with exposure to these two biocidal agents, but we also explored the minimum concentration of each that is necessary to affect the virus structure.

**Figure 3:**
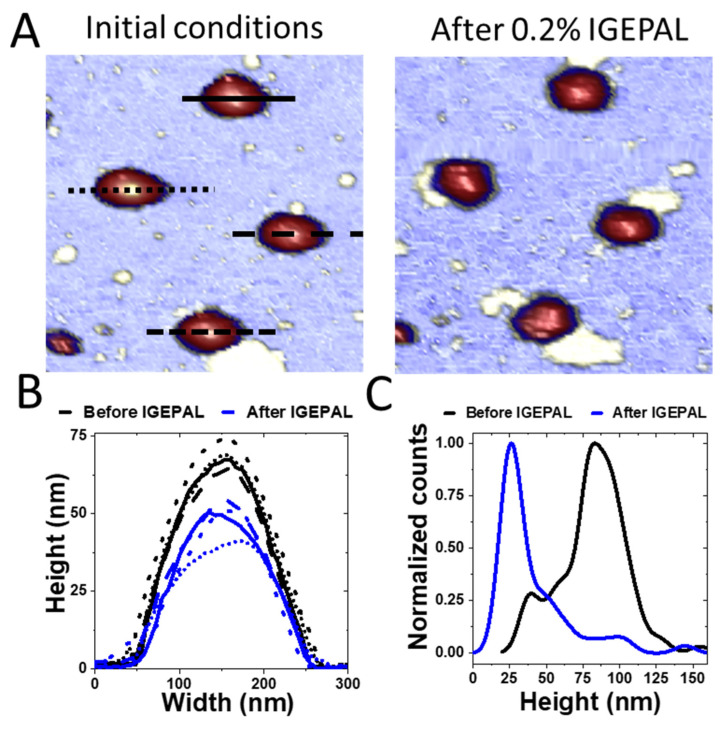
Treatment of TGEV with IGEPAL 0.2% (**A**). Topographical images before (left) and after (right) IGEPAL treatment (**B**). Profiles traced over the particles before (black) and after (blue) the treatment. The time interval between images was ~30 s (**C**). Height distribution of TGEV particles before (black) and after (blue) treatment (*n* = 103). Counts taken from the distribution curve were normalized for comparison. The peak shifts from the value of the intact particle height to the height of the cores.

**Figure 4:**
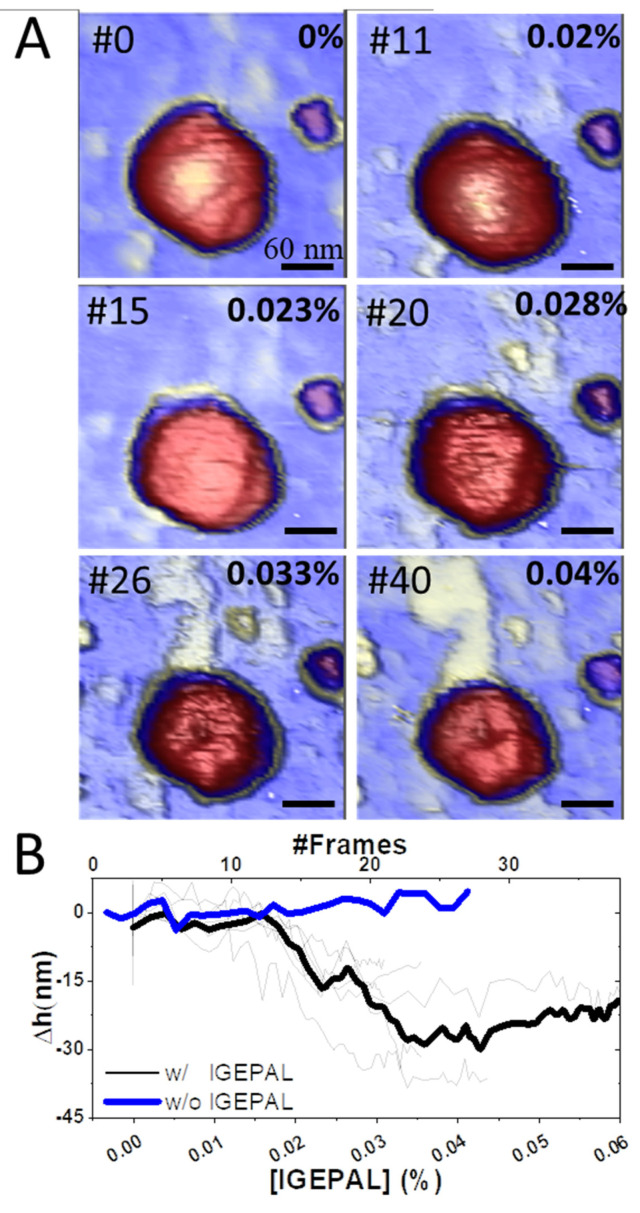
Effect of IGEPAL gradient on TGEV virions (**A**). Topographical images were taken while the amount IGEPAL was increased (Video S4). The frame number is shown in the upper left corner and the concentration of IGEPAL in the right (**B**). Height loss during the time course assay. Experimental curves are shown in grey and the average curve of 8 observations is shown in black. As a control, the height of 3 particles (Video S5) was tracked in buffer without detergent (blue).

In the case of detergents, we used an anionic, non-denaturing detergent (IGEPAL-CA-630) to test in situ the effect of mild surfactants on the viral particle. IGEPAL has been extensively used in biochemistry for solubilizing the membrane of CoVs without affecting viral proteins [50,59,60]. In addition, this agent has been also used as a viral disinfectant [9,61,62]. Therefore, IGEPAL is a representative agent of anionic detergents such as Triton x-100 and Tween 20 whose mild strength activity could be extrapolated to strong agents such as SDS. Once the viruses were adsorbed on the surface (Figure 3A, left), we added IGEPAL at 0.2% (*v*/*v*) in the AFM liquid cell and monitored the individual virus structure afterwards (Figure 3A, right). Measuring the particle height before (Figure 3B, black) and after (Figure 3B, blue) showed a decrease of height that could be ascribed to the removal of structural lipids. In fact, a statistical analysis of 103 particles indicated that initially, most virion heights (~80 nm) were compatible with intact TGEV structures (Figure 3C, black). After IGEPAL treatment, most TGEV particles reduced their height to ~27 nm (Figure 3C, blue), although some of them were still intact (~5%). These results informed about the initial and final states of TGEV particles but lacked information about gradual virus deterioration in the presence of increasing IGEPAL concentration over time. To investigate this process in detail, we took consecutive images of the same TGEV virus particle while increasing IGEPAL concentration (Figure 4A) in the liquid cell [63]. One virus was continuously imaged more than 40 times while IGEPAL was increased up to ~0.06% for 80 min (Video S4). The virus particle remained stable at 0.020% IGEPAL, although at 0.023% some deterioration could be recognized. In frame #26, it was possible to observe some material ejected from the virus and a circular crack at the top. This damaged structure seems stable until frame #40. For statistical analysis, we monitored eight particles subjected to similar processes and plotted their individual (Figure 4B, thin grey) and average (Figure 4B, thick dark) height variation over time. It is known that the AFM imaging process itself induces the disruption of some viruses, such as lambda phage and human adenovirus [31,64]. To rule out that effect as being responsible for the decrease in height, consecutive images of virions in buffer without detergent were taken as control (Figure 4B, blue, Video S5), showing that their heights were not affected by AFM scanning.

**Figure 5:**
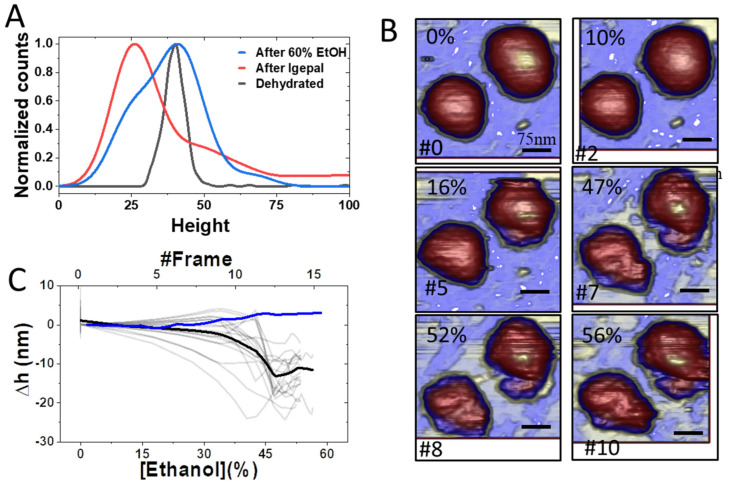
Effect of ethanol gradient on TGEV virions (**A**). Comparison of virion height distribution after treatment with 60% ethanol, 0.2% IGEPAL or dehydration (**B**). Consecutive imaging of TGEV virions during the increase in ethanol concentration. The concentration of ethanol is shown in the upper left corner and frame is shown in the lower left corner (Video S6) (**C**). Track of the height loss during the ethanol time course. Experimental data is shown in grey, the average curve of 23 observations is shown in black and the average of 5 viruses without ethanol is shown in blue (Video S5).

Likewise, to study the effect of ethanol on viral particles, particle heights before and after treatment with 60% ethanol (*v*/*v*) were explored (Figure 5A). Ethanol-treated virions followed a height distribution similar to that of the dehydrated particles, plus a shoulder at 27 nm corresponding to the peak shift when virions were treated with IGEPAL (Figure 5A). To explore the gradual effect of ethanol on virus topography in real time, we monitored the structural changes of individual TGEV virions while increasing the ethanol concentration in the AFM liquid cell. Figure 5B shows the simultaneous evolution of two nearby virus particles that started showing deterioration at 16% ethanol concentration (Video S6). This degradation accelerated as the ethanol concentration increased to 47%, resulting in evident cracks occurring above 47% until the final concentration of 56% was reached. For statistical analysis, these experiments were carried out on 23 virus particles, and in all cases, there was an evident deterioration of the particles as the ethanol concentration was increased (Figure 5C, thin grey). The averaged behavior (Figure 5C, thick black) reveals that the viral structures stabilize after losing around 10 to 20 nm in height.

## 5. Discussion

The goal of our experiments was to explore the mechano-structural stability and behavior of TGEV (as a surrogate for SARS-CoV-2) in a variety of harsh physicochemical environments. Although high resolution tomography (Figure 1A) of TGEV reveals spikes that are structurally similar to those of SARS-CoV-2 [65], our observations of TGEV virions of the same purification in liquid milieu revealed smooth surfaces (Figure 1B). This effect has also been described for SARS-CoV-2, and has been attributed to the rapid movement of the spikes (Kiss et al. 2021) [16]. We believe that another possibility is that the spikes yield under the AFM tip force (~80 pN), reaching the viral capsid with the apex of the AFM stylus. This effect has also been recently found in human adenovirus [64], where the fibers remained invisible to AFM due to their great flexibility, despite their large size. However, fibers can be imaged when they are short and thick and robustly bonded to the capsid, as is the case of human rotavirus, where spikes can be visualized over a few frames before being wiped out during the imaging process [28]. In fact, this other possibility cannot be completely neglected in TGEV because AFM images show some debris around the virus particle (Figure 1B, blue triangles) that may correspond to disrupted spikes. In the case of SARS-CoV-2, a recent study combining cryo-electron tomography and simulations determined that the spike was highly flexible and mobile through the hinge region that connects it to the membrane [65]. This flexibility, combined with the low density of S protein on the virus surface (~20 spikes) [65], results in the spikes spanning ~30% of a typical AFM tip of 12 nm in radius. This estimation indicates that the AFM tip apex is most probably mapping the membrane of the virus and not the extent of the S layer.

**Figure 6:**
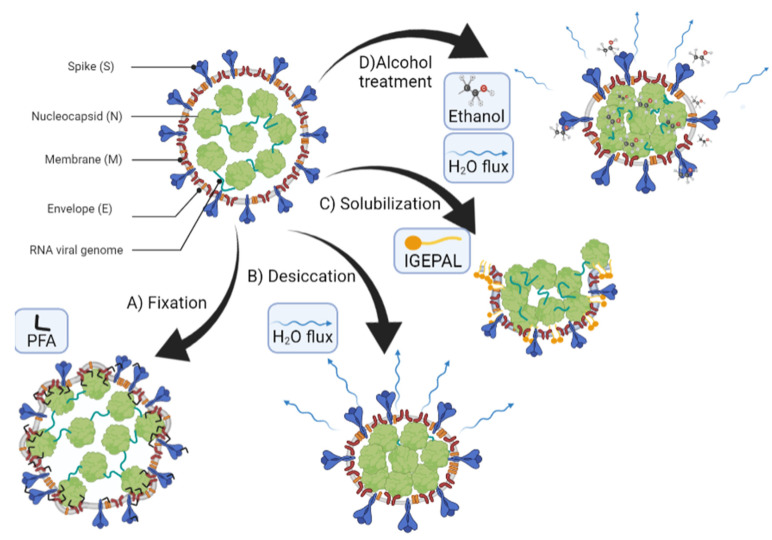
Proposed effects for the different environmental conditions. Paraformaldehyde (PFA) treatment, which fixes the RNPs and membrane proteins to the capsid layer. Desiccation of the viral particle reduces the virion size. IGEPAL solubilizes the membrane lipids while uncovering the core and allows some RNPs to spread out around the virion. Alcohol induces both dehydration and loss of material.

Fixation of native virus structures with PFA resulted in rugged structures (Figure 1D) with an average height of 80 nm (Figure 1G). Although the change from smooth to wrinkled surfaces after chemical fixation has been previously ascribed to the spikes [16], we suggest that the fixation process fits the virus envelope to the subsurface RNP structures [66] (Figure 6), probably via the M protein, which not only is the most abundant structural protein, but also has been proven to interact with the RNP-genome [67]. In fact, this behavior was repeated in the comparison of dehydrated native and dehydrated PFA-fixed particles, where the non-fixed ones had a smooth surface (Figure 1C) and the fixed ones had a rough surface (Figure 1F). As for the height, dehydration of unfixed virions produced a clear decrease in its mean height, as has been observed in other viruses, whose collapsed structures had lost water and the proteins had been partially denatured [55]. Further rehydration of native TGEV particles (Figure 1D) recovered the particles’ height (Figure 1G) although with some rugosity on the surface that could correspond to the denatured proteins in the membrane. However, dehydration during the staining process for negative stain electron microscopy does not disrupt other viral components, such as the lipid membrane, RNPs or the spike complex (Figure S4) that are key in the infection cycle as the virus is able to keep high levels of infectivity [20,68]. 

Beyond the intrinsic pleomorphism of enveloped viruses, the broad height distribution of native TGEV (Figure 1G) is probably also related to the deformation of virus particles upon surface adsorption [53] and the packing of genetic material inside the virus. Specifically, CoVs pack non-segmented, positive-sense RNA molecules of 25–32 kb within the ~80 nm diameter viral cavity. Although the TGEV core is still not resolved with sufficient resolution, structures of other CoVs [66,69,70] show a common ‘pearl necklace’ organization of RNPs. In SARS-CoV-2, the genome is packed in RNPs grouped in hexameric and pyramid forms depending on the virion shape [66]. Groups of N proteins and the RNA form spherical particles (RNPs) assembled as ‘eggs-in-a-nest’, tightly packed in the virus internal lumen but leaving room between each other. However, the ‘pyramid’ assembly is more prone to happen in ellipsoidal viruses, in which the RNPs solidly fill the virus cavity [66]. In this context, native TGEV particles with heights ~80 nm (Figure 2D and Figure S1B) would correspond to spherical viruses packing RNPs in ‘eggs-in-a-nest’ assembly, whereas particles with heights ~50 nm would ascribe to more compactly filled ellipsoidal viruses. In fact, it is possible to observe in the TGEV tomogram big particles with empty room inside (Figure S1A, black arrow) and small particles with the cavity fully packed with RNPs (Figure S1A, blue arrow).

We can correlate our mechanical results with these two assemblies. Deformation of indentation-resistive virions presented a drop in the normal force between 0.1 nN and 0.3 nN (Figure 2E and Figure S2). This drop of the normal force usually corresponds to a fragile break of the virus structure, although no evident damage was inflicted to the viral particles. The same resilient behavior extended to consecutive indentation-imaging cycles of the viruses (Figure 2C, black). This unrelenting stability of TGEV virions points to the fact that the lipid envelope was not being affected [71]. Force steps (Figure 2E and Figure S2A) might correspond to the shifting and accommodation of underlying RNPs under the AFM tip to mechanical stress as it happens in other virus systems [72]. By assuming that all viruses package the same amount of genome, it is likely that everyone contains a similar number of RNPs, no matter their size. Therefore, the larger height of these particles (Figure 2D) suggests a loosened core state represented by the ‘eggs-in-a-nest’ assembly. In this case, when the tip retracts, RNPs recover their original positions and the flexible membrane returns to its previous height. Even though there was no structural damage, the particles had a dynamic behavior when indented (Video S2), reinforcing this idea of RNP rearrangement. Structural data [66] point to a low packing fraction (~14%) that would facilitate these rearrangement processes. Similar shell-like, hollow protein cage structures can relax the mechanical strain, with the ability to yield and recover after nanoindentation [73,74]. Indeed, consecutive nanoindentations on the same virus presented a similar pattern (Figure S2A), indicating that the nucleoproteins inside could reorganize after each indentation in a similar way. Our mechanical data agrees with the SARS-CoV-2 results (Kiss et al. 2021), with similar spring constants (Figure S3) and force drop, further indicating that TGEV is an excellent surrogate model for this type of study. From the force-strain curves we learned that indentation-resistant virions (Figure 2E) undergo a homogeneous deformation until 0.1 nN, but beyond this point show a heterogeneous behavior which is probably related to the pleomorphism of the viruses and the loose architecture of the RNPs mentioned above.

In contrast, indentation-sensitive particles exhibited lower heights than the resistive ones (Figure 2D) and showed a homogeneous behavior throughout the whole indentation experiment (Figure 2F). The normal force began to increase almost linearly from 0 indentation, but at ~0.15 nN, when the particles had been indented about 50%, there was a conspicuous drop of the normal force (~0.1 nN). These data indicate that the virus particles were elastically deformed until cracking open with a permanent fracture (Figure 2B) of ~25 nm in depth (Figure 2C, blue). After this fracture, the tip could penetrate into the nucleocapsid core which was deformed in a Hertzian like fashion (Figure 2F), as happens in other viruses when probing the core [75]. The lower height indicates a higher packing fraction (‘pyramid’ packing) [66] without internal free room, preventing the RNPs from rearranging under the mechanical stress. In fact, structural data of SARS-CoV-2 indicate this ‘pyramid’ packing occupies ~36% of the cavity, very close to viruses with high packing fraction values, such as lambda and phi29 bacteriophages [76]. Together with the AFM tip, solidly packed RNPs act as a “hammer and anvil” on the membrane, concentrating the mechanical stress on the virus envelope and causing permanent fractures (Figure 2B). Consecutive imaging of the virus particles after each nanoindentation (Figure 2B) reported increasing damage, with virus height loss from 25 to 100 nm (average decrease ~40 nm; Figure 2C, blue).

It is worth noting the different behavior of FDC after the drop in force presented in Figure 2E,F. Usually, after brittle viruses are broken, the AFM stylus penetrates into the cavity, like cracking an eggshell. In particles with a low packing fraction, mechanical resistance is low and the cantilever experiences a free fall into the capsid until making contact with the substrate [77]. The surface of the substrate, which is much harder than the viruses, does not yield, and the increase in normal force results in a quasi-vertical line at a strain close to 1 for virions with large diameter (Figure 2E). Conversely, if the tip finds a tightly packed environment that cannot shift under the mechanical stress induced by the tip, a Hertzian progressive increase in the normal force takes place [75], as occurred in the indentation-sensitive viruses (Figure 2F) from 0.5 stress to 1.

Beyond mechanical stress, the effects of chemical treatment on TGEV particles were also deeply related to the virion structure. Our experimental data indicate that virions exposed to 0.2% IGEPAL quickly lost their membranes (Figure 3 and Figure 6). Data prior to IGEPAL treatment (Figure 3C, black) showed that most virions had a height of 80 nm (intact particles), although there was a small shoulder at 30 nm that could be ascribed to free cores [19]. After IGEPAL addition, the height of the main population shifted from ~80 to ~27 nm, very similar to this presumed free core population (Figure 3C, blue). It has been shown that Triton X−100 promotes membrane solubilization and core release [78]. In those cases, the core could remain as a compact or elongated structure, such as those that appeared upon IGEPAL treatment (Figure 3A, right; Figure 4A, #26 yellow; SV4). Interestingly, ethanol seemed to induce two effects on TGEV: dehydration and disruption. This dual effect can be observed in Figure 5A, where the height of the main virus population (~40 nm) after treatment with 60% ethanol (blue) is almost identical to the height of native viruses in a dehydrated state (black). It is important to remark that virion dehydration does not necessarily imply the loss of the spikes. In fact, the process of negative staining in standard electron microscopy results in virion dehydration, but the spikes remain clearly visible (Figure S4). Heights corresponding to cores (~30 nm) appear as a tiny shoulder that coincides with the virus height after IGEPAL treatment (red). IGEPAL and ethanol time courses caused structures to protrude from the surface of some virions (Figure 3A, Figure 4A, Figure 5B and Figure S5C). The topography of the structures measured by AFM resembled those imaged by transmission electron microscopy obtained after treatment of virions with NP40, a detergent equivalent to IGEPAL [78], which supports them being RNPs. The amount of detergent needed to start virus disruption was very low compared to ethanol. In particular, viruses began to lose material at an IGEPAL concentration of 0.02% (Figure 4B, black), and they seemed to stabilize after losing between 15 nm and 30 nm in height. This variability is likely related to the pleomorphism of TGEV. Altogether, these experiments show that IGEPAL is a much more effective antiviral than ethanol, which is relevant when considering effective and sustainable sanitizing and hygienic procedures involving any CoV, including SARS-CoV-2. In general, that use of soap/detergent is very efficient to disrupt enveloped viruses making hand washing/cleaning with soap/detergents a more efficient way than ethanol-based disinfection.

## 6. Conclusions

In this work we have elucidated the mechano-structural properties of TGEV and the way in which individual virion structures are altered in situ while varying the presence of virucidal agents gradually. We showed that while larger TGEV particles can withstand consecutive mechanical assaults, smaller structures are prone to physical breakdown under the same conditions. Our experiments in real time revealed that the structure of TGEV virions is disrupted by detergent concentrations three orders of magnitude lower than ethanol. IGEPAL was able to reduce virus height more than ethanol (~15–30 nm vs. ~12 nm). These two facts likely indicate that the infectivity would be more affected when using detergent than ethanol. TGEV’s mechanical properties appear very similar to those of SARS-CoV-2, and the structural similarity between them shows the utility of surrogate virus models as an alternative strategy for investigating highly infectious viruses which require BSL3 laboratory facilities. This work may help optimize sanitizing protocols in both daily life and in industrial processes. Various antiviral elements are currently being used in disinfection at all scales, whether for personal use or on an industrial scale, and they entail a cost overrun that can be alleviated by adjusting the concentration to the one which is strictly necessary.

## Data Availability

Not applicable.

## Supplementary Materials

The following are available online at https://www.mdpi.com/article/10.3390/cells11111759/s1, Figure S1: A. AFM topographical image of a typical TGEV sample; Figure S2: A. FDC curves performed on indentation-resistant virions; Figure S3: A. Mechanical properties in the indentation-resistant and indentation-sensitive virions; Figure S4: Negative staining electron microscopy image of TGEV; B. Table showing the numerical values of these properties; Figure S5: A. Consecutive AFM topographical images of a single virus particle after two indentations. Inset shows the height profiles. B,C. Gallery of images after IGEPAL (C) and ethanol (D) treatment, Video S1: This video shows the cryotomogram sections of a purified TGEV sample used in the AFM assays; Video S2: It shows the effect of performing consecutive nanoindentations on a resistant TGEV virion; Video S3: This video shows the effect of performing consecutive nanoindentations on a sensitive TGEV virion; Video S4: It shows the effect of adding IGEPAL at a concentration of 0.08% *v*/*v*; Video S5: Fatigue assay of TGEV in buffer, by taking 46 frames at a constant force of 100 pN during 80 min; Video S6: This video shows the effect of ethanol on a single virion.
