# Supplementary material for: Monitoring SARS-CoV-2 Surrogate TGEV Individual Virions Structure Survival under Harsh Physicochemical Environments
Source: Cells. 2022 May 27;11(11):1759. doi: 10.3390/cells11111759 (PMC9179875; doi:10.3390/cells11111759)
Supplement: Supplementary file 1 [file cells-11-01759-s001.zip › cells-1703387-supp/cells-1703387-supp.pdf]

Monitoring SARS-CoV-2 surrogate TGEV individual virions structure survival under harsh physicochemical environments

M. Cantero, D. Carlero, F. J. Chinchón, J. Martín-Benito and P. J. de Pablo

Supplementary Figures

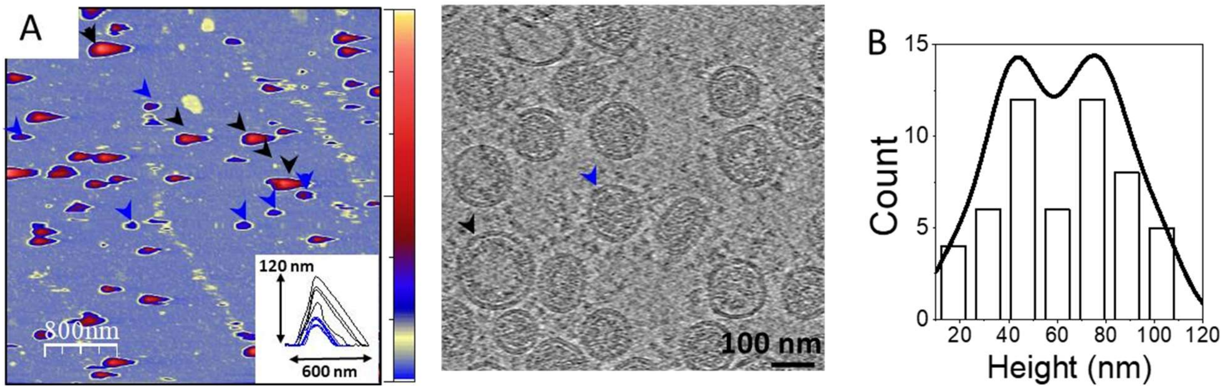

Figure S1. A. AFM topographical image of a typical TGEV sample (left). Black and blue arrows pointto representative virions of large and small size populations, respectively. Transmission electron microscopy image captured from the tomogram of video SV1 (right). B. Height distribution of the AFMimage. Two populations can be resolved, corresponding to the black and blue profiles of Fig. S1A inset.

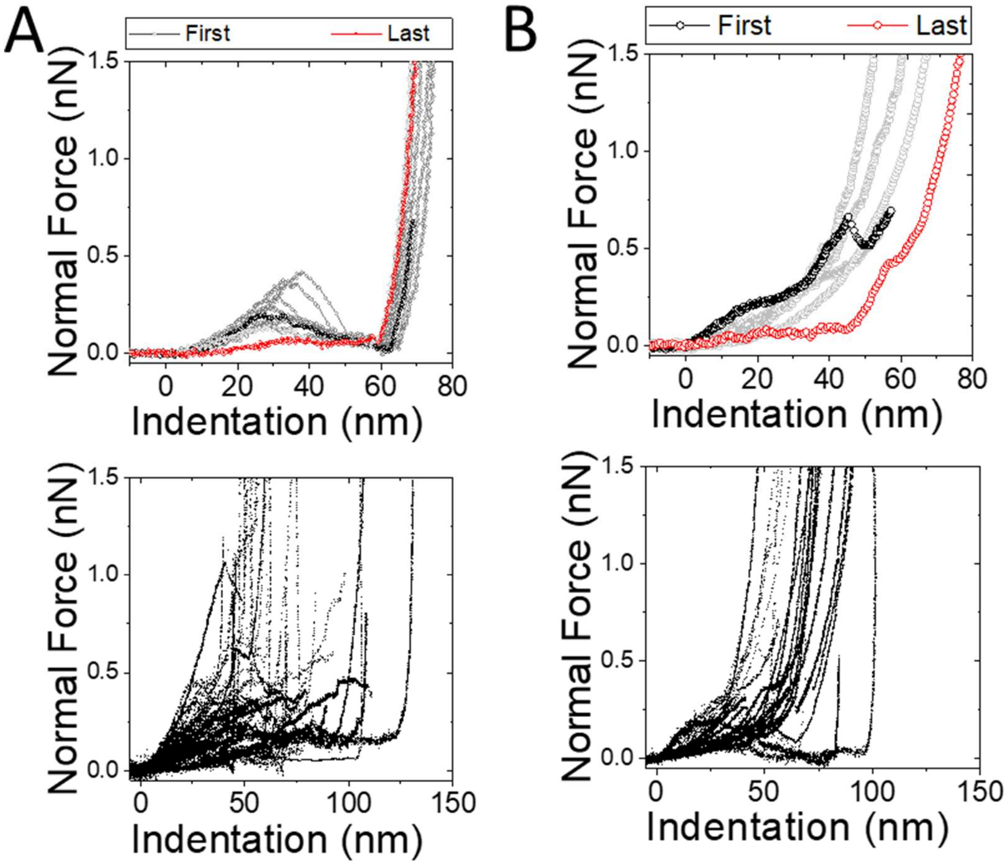

Figure S2. A. FDC curves performed on indentation-resistant virions. Upper panel shows consecutiveexperiments performed on the same virus (black first, red last, grey the rest). Lower panel shows allperformed FDC of indentation-resistant virions, whose strain is shown in Fig. 2E. B. FDC curves of indentation-sensitive virions. Upper panel shows consecutive experiments performed on the same virion (black first, red last, grey the rest). Lower panel shows all FDC performed on indentation- sensitive virions, whose strain is shown in Fig. 2F.

A

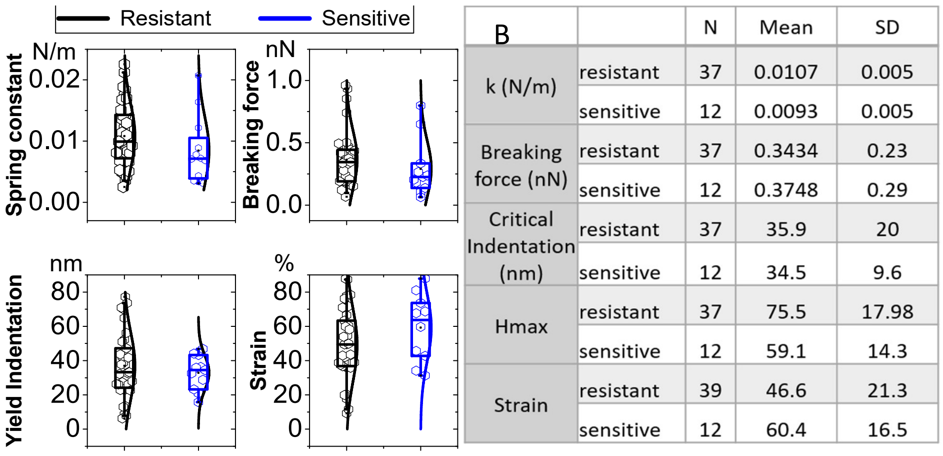

B

		N	Mean	SD
k (N/m)	resistant	37	0.0107	0.005
	sensitive	12	0.0093	0.005
Breaking force (nN)	resistant	37	0.3434	0.23
	sensitive	12	0.3748	0.29
Critical Indentation (nm)	resistant	37	35.9	20
	sensitive	12	34.5	9.6
Hmax	resistant	37	75.5	17.98
	sensitive	12	59.1	14.3
Strain	resistant	39	46.6	21.3
	sensitive	12	60.4	16.5

Figure S3. A. Mechanical properties in the indentation-resistant and indentation-sensitive virions. B.Table showing the numerical values of these properties

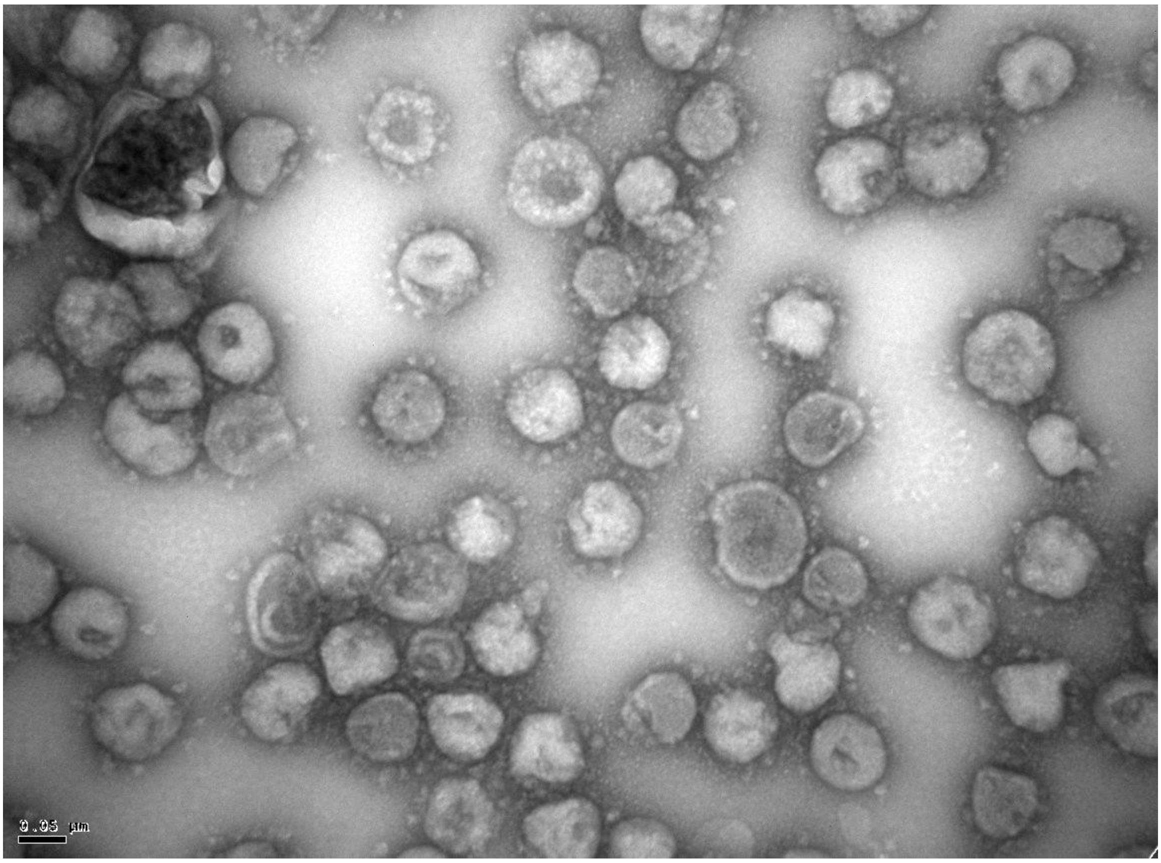

Figure S4. Negative staining electron microscopy image of TGEV.

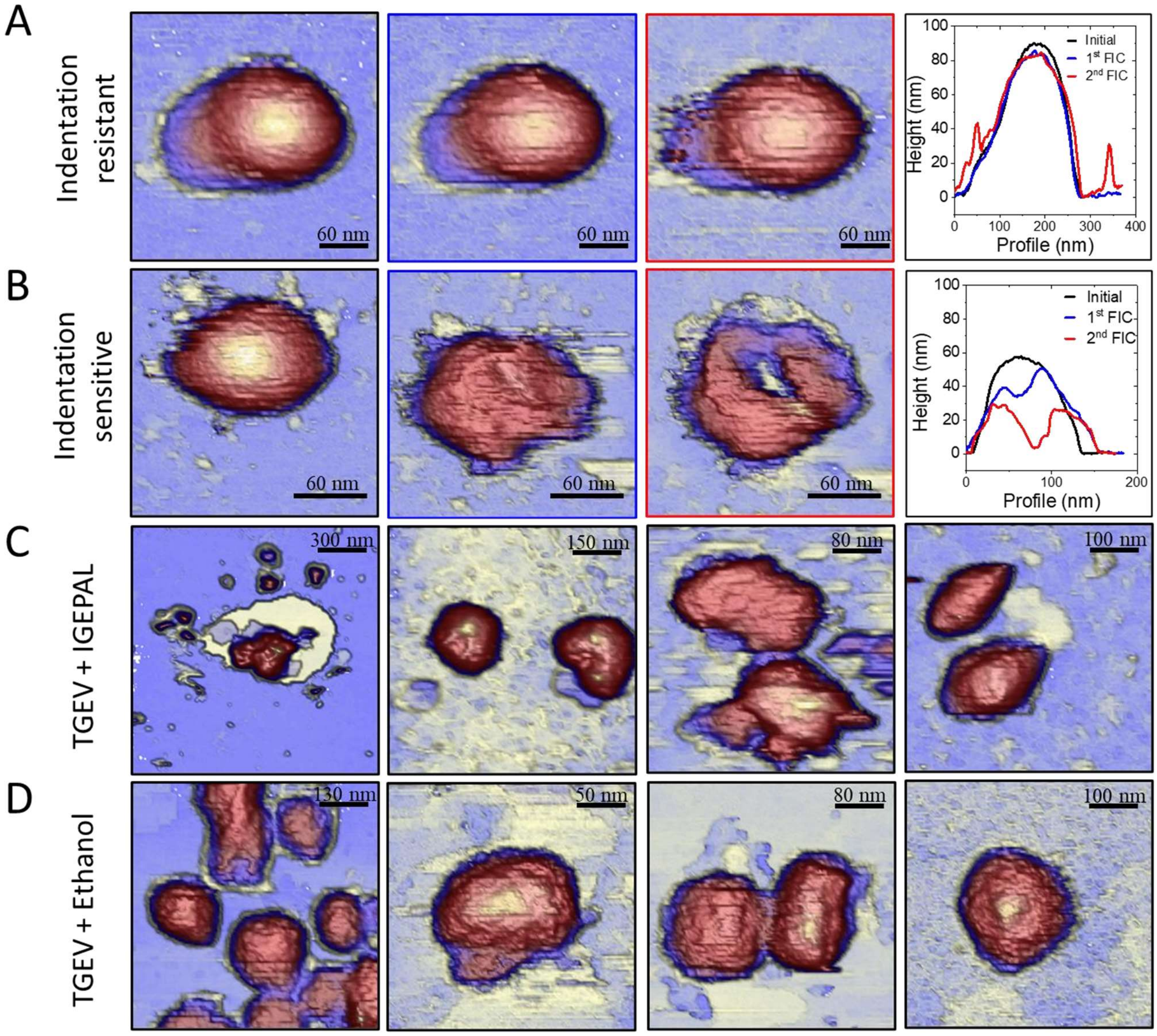

Figure S5. A. Consecutive AFM topographical images of a single virus particle after two indentations.Inset shows the height profiles. B,C. Gallery of images after IGEPAL (C) and ethanol (D) treatment.

Supplementary Videos

Video S1. This video shows the cryotomogram sections of a purified TGEV sample used in the AFMassays. The spikes, the envelope membrane and the RNPs inside the virion are clearly visible. The pleomorphism of the virus is also evident showing virions of different sizes. This video corresponds to Figure 1A.

Video S2. It shows the effect of performing consecutive nanoindentations on a resistant TGEV virion. A single indentation is done before each frame. The images were acquired at a constant forceof 100pN. The video is composed of 8 frames taken during 38 min. This video corresponds to Figure2A.

Video S3. This video shows the effect of performing consecutive nanoindentations on a sensitive TGEV virion. A single indentation is done before each frame. The images were acquired at a constantforce of 100pN. The video is composed of 8 frames taken during 38 min. This video corresponds to Figure 2B.

Video S4. It shows the effect of adding IGEPAL at a concentration of 0.08% v/v. The total number of frames is 18, with an elapsed time of 60 min. The scanning force is 60 pN. This video correspondsto Figure 4.

Video S5. Fatigue assay of TGEV in buffer, by taking 46 frames at a constant force of 100 pN during 80 min.

Video S6. This video shows the effect of ethanol on a single virion. Ethanol was flowed onto the sample up to a final concentration of 40%. A total of 46 frames were acquired at a constant force of 150 pN during 3h and 10 min. This video corresponds to Figure
